# Phenotypic differentiation of gastrointestinal microbes is reflected in their encoded metabolic repertoires

**DOI:** 10.1186/s40168-015-0121-6

**Published:** 2015-11-30

**Authors:** Eugen Bauer, Cedric Christian Laczny, Stefania Magnusdottir, Paul Wilmes, Ines Thiele

**Affiliations:** Luxembourg Centre for Systems Biomedicine, University of Luxembourg, Esch-sur-Alzette, Luxembourg

**Keywords:** Genome-scale metabolic reconstructions, Metabolic modeling, Metagenomics, Intestinal microbiota, Evolution, Ecology

## Abstract

**Background:**

The human gastrointestinal tract harbors a diverse microbial community, in which metabolic phenotypes play important roles for the human host. Recent developments in meta-omics attempt to unravel metabolic roles of microbes by linking genotypic and phenotypic characteristics. This connection, however, still remains poorly understood with respect to its evolutionary and ecological context.

**Results:**

We generated automatically refined draft genome-scale metabolic models of 301 representative intestinal microbes in silico. We applied a combination of unsupervised machine-learning and systems biology techniques to study individual and global differences in genomic content and inferred metabolic capabilities. Based on the global metabolic differences, we found that energy metabolism and membrane synthesis play important roles in delineating different taxonomic groups. Furthermore, we found an exponential relationship between phylogeny and the reaction composition, meaning that closely related microbes of the same genus can exhibit pronounced differences with respect to their metabolic capabilities while at the family level only marginal metabolic differences can be observed. This finding was further substantiated by the metabolic divergence within different genera. In particular, we could distinguish three sub-type clusters based on membrane and energy metabolism within the *Lactobacilli* as well as two clusters within the *Bifidobacteria* and *Bacteroides*.

**Conclusions:**

We demonstrate that phenotypic differentiation within closely related species could be explained by their metabolic repertoire rather than their phylogenetic relationships. These results have important implications in our understanding of the ecological and evolutionary complexity of the human gastrointestinal microbiome.

**Electronic supplementary material:**

The online version of this article (doi:10.1186/s40168-015-0121-6) contains supplementary material, which is available to authorized users.

## Background

Recent advances in sequencing technologies have greatly improved our knowledge about the metabolic complexity of the human microbiome and provide novel approaches to identify beneficial microbes [1]. In particular, sequencing the (ideally) entire genomic content (i.e., metagenomic sequencing) of the intestinal microbiota has allowed the establishment of a catalog of main groups of microorganisms present in the gastrointestinal tract and potential metabolic pathways [3] by avoiding culturing and isolation of individual microbial organisms. In this respect, endeavors of the human microbiome project [4] and the MetaHIT consortium [5] aim at establishing comprehensive data-sets of metagenomic content, metabolic functions, and taxonomic compositions within human individuals as well as the isolation and sequencing of numerous microbial taxa.

Despite these efforts, however, we are still lacking a comprehensive mechanistic understanding of the intestinal microbiota. One major hurdle in achieving this goal is the lack of organismal system boundaries, enabling us to associate the presence of metabolic pathways in the microbiome with a specific bacterium. Inferring metabolic roles by taxonomic classification alone is difficult because phylogenetically closely related organisms might be very different in their metabolism [6]. It may be therefore challenging to associate functional roles to entire taxonomic groups [7] to conjecture the biological relevance of intestinal bacteria. For instance, members of the same genus, or even of the same species, can be both probiotic and pathogenic [8], indicating a differential strain-specific adaptation. In this context, nutrient utilization can be a strong determinant for the adaptation to varying environments, since it can give a competitive advantage to other organisms that are metabolically less versatile. Thus, having additional metabolic functions can aid microbes in occupying further niches within the human gut. Accordingly, the functional consequences for the host change.

Current developments in systems biology allow the modeling of microbial metabolism to gain a mechanistic insight into the relationship between genotype and phenotype [9]. Genome-scale metabolic reconstructions form the basis of such modeling efforts. A reconstruction is assembled based on the genomic sequences as well as biochemical and phenotypic data of a target organism, and accounts for metabolic genes, enzymes, and their associated reactions [10, 11]. Genome-scale metabolic reconstructions serve as a blueprint for condition-specific metabolic models [10, 11], which are obtained by the application of constraints, such as known nutrient uptake rates. The reconstruction process often includes a gap-filling procedure [12, 13], in which additional reactions are included to better model biologically relevant phenotypes, such as the formation of all known biomass precursors [14]. Metabolic models can be studied using a variety of mathematical methods [15]. One frequently used approach is flux balance analysis, which is applied to investigate a functional steady-state flux distribution of the modeled system, while maximizing (or minimizing) a particular cellular objective (e.g., production of biomass precursors) [10]. This modeling approach has been used to investigate nutrient requirements [16], gene essentialities [17], and metabolic interactions [18] for organisms of interest, thereby providing new insights into phenotypic and metabolic properties. The reconstruction process relies on the availability of detailed phenotypic data for the target organism [11], which is usually not available for many of the commonly found microbes in the human gut [1, 3]. To obtain representative metabolic reconstructions for these less well-studied organisms, automatic tools have been developed in recent years, such as the Model SEED platform [19], to provide a valuable starting point for metabolic modeling. In fact, draft reconstructions have been used to generate hypotheses about the target organisms with subsequent experimental validation, leading to the refinement of the metabolic reconstruction [14, 20–22].

In this study, we generated automatically refined draft genome-scale metabolic models of 301 representative intestinal microbes in silico based on whole genome sequences of the human microbiome project using an established approach [19]. We applied a combination of unsupervised machine-learning and computational modeling techniques to study individual and global differences of the metabolic models and the original genomes. Our key results include: i) divergent reactions involved in energy metabolism and membrane synthesis which are most relevant to discriminate different phylogenetic groups, ii) a linear relationship between differences in metabolic reaction potential and essential nutrients determined by flux balance analysis which indicates that the phenotype is directly correlated to the metabolic repertoire, iii) differences in metabolic reaction potential and phylogeny which exhibit an exponential relationship, suggesting an explanation as to why closely related microbes can be very different in their metabolic traits while at less-resolved phylogenetic distances only marginal differences in metabolic diversity can be observed, iv) local differences in pathway presence which can be used to further distinguish representatives of *Lactobacillus*, *Bifidobacteria*, and *Bacteroides*. In summary, we demonstrate the importance of the metabolic repertoire of microbes to predict their phenotypic behavior in an ecological and evolutionary context.

## Results and discussion

### Selected microbes as a model for the human gut microbiota

**Fig. 1:**
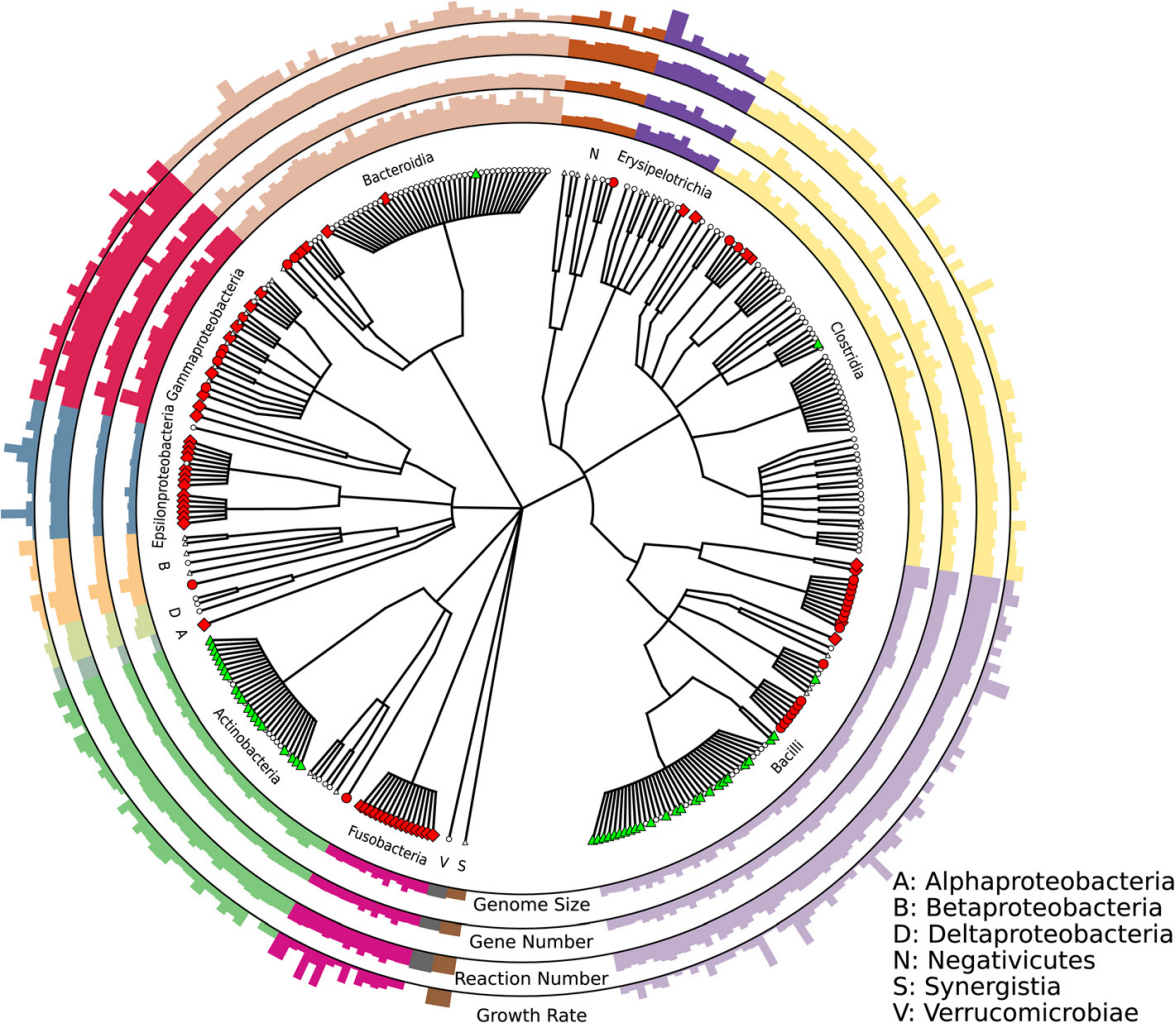
Phylogeny and individual statistics of the microbe selection. The cladogram shows the taxonomic relationships among the 301 microbes. In the four outer layers, the *bars* represent the relative individual genome size, number of genes, number of reactions, and *in* silico growth rate. The different *colors* represent the various bacterial classes. The *leaf colors* and *shapes* symbolize whether a microbe is a probiotic (*green triangle*), a pathogen (*red diamond*), an opportunistic pathogen (*red circles*), or a non-pathogenic bacterium (*white triangles*)

In order to answer ecological and evolutionary questions relevant for human health and disease, we selected 301 commonly found gut microbes based on their reported occurrence in the healthy gut microbiome [1, 3] and the availability of sequenced isolate genomes (Fig. 1). We used the Model SEED platform [19] to generate automated draft genome-scale metabolic reconstructions for each microbe. To enable growth under anaerobic conditions, which are predominant in the human gut [23], we added specific reactions, if necessary (Additional file 1: Table S2). A comparison of our draft reconstructions with a set of published manually refined high-quality metabolic reconstructions taken from [24] revealed that most of the metabolic functionalities were captured in the refined draft reconstructions (Additional file 2: Figure S1). Reactions absent in the refined draft reconstruction belonged mostly to the category of transport and exchange reactions, whose addition requires experimental and physiological data, as substrate specificity and transport mechanism is difficult to automatically annotate in microbial genomes [25].

Our set of refined draft reconstructions captured a wide spectrum of different phyla (Fig. 1) with a taxonomic diversity similar to what is commonly observed in the human intestine [3]. The high diversity and proportion of microbes within the phyla Bacteroides, Proteobacteria, and Firmicutes (Fig. 1) is concordant with observations in the human colon [26]. Moreover, by integrating information about pathogenic and beneficial traits of each microbe (Fig. 1), we were able to associate these metabolic traits with the phenotype toward the host. As expected, a large proportion of probiotic *Lactobacillus* and *Bifidobacteria* could be found in the classes Bacilli and Actinobacteria, respectively (Fig. 1 and Table 1). Additionally, known pathogenic organisms within the Proteobacteria, Fusobacteria, and Bacilli are also represented. Thus, our selection of bacteria provides an appropriate representation of microbial species, phenotypic traits, and metabolic processes present in the colon, the main site of microbial fermentation and interaction of microbes with the host [27].

**Table 1:** Genome and metabolic model statistics of the selected microbes

		Average per taxonomic group				
Taxon	Number models	Genome (Mbp)	Genome complete^a^	Number genes	Number reactions	Gap-filled reactions^b^
All taxa	301	3.3	95 %	702	875	3 %
Class						
Bacilli	68	2.5	95 %	656	860	4 %
Clostridia	61	3.5	96 %	735	839	1 %
Bacteroidia	51	5.3	95 %	764	908	1 %
Actinobacteria	36	2.3	95 %	514	727	10 %
γ-Proteobacteria	25	4.6	95 %	1127	1311	1 %
Genus						
*Lactobacillus*	37	2.3	96 %	597	798	4 %
*Bifidobacterium*	29	2.2	95 %	505	734	12 %
*Bacteroides*	43	5.6	96 %	774	909	1 %

^a^Based on a selection of 107 essential genes [73]

^b^Based on the total number of reactions in the model. Gap-filling reactions were mostly added by the Model SEED platform

Our analysis also included microbes with draft genomes (Additional file 3: Table S1), requiring the assessment of the overall genome completeness and the potential impact on gene annotations and consequently on the generated metabolic reconstructions. The completeness and possible genomic contamination by other microorganisms of the individual 301 of the individual genomes was assessed using a collection of 107 universal, single-copy genes [28, 29]. In our set of 301 genomes, the average estimated genome completeness was 95 % (Table 1). We further investigated the genome size and annotated genes among the 301 organisms (Table 1). Gammaproteobacteria had generally large genomes and a high number of annotated genes, while members of the order Bacteroidia had in general larger genomes but a lower number of annotated genes. This difference could be attributed to differences in annotation efficiencies, as Proteobacteria (in particular, gut specific *Escherichia* species) are very well-studied and thus have more homologous genes. Consequently, the number of reactions in the constructed metabolic models was higher and the number of reactions added via gap-filling lower. In contrast, we found a higher number of gap-filled reactions and a lower number of reactions in Actinobacteria (Table 1). This bias, which is well established for metagenomic analyses [30], is most likely the result of having less experimental data and validated gene annotation available for Actinobacteria. The presence of this apparent annotation bias underlines the limitation in current annotation techniques affecting particularly phylogenetically distant microbes [29–31] and highlights the need for more detailed experimental biochemical studies to elucidate gene functions in phyla distant to those containing model organisms [31].

### Global reaction differences recapitulate conserved taxonomic patterns and phenotypes

**Fig. 2:**
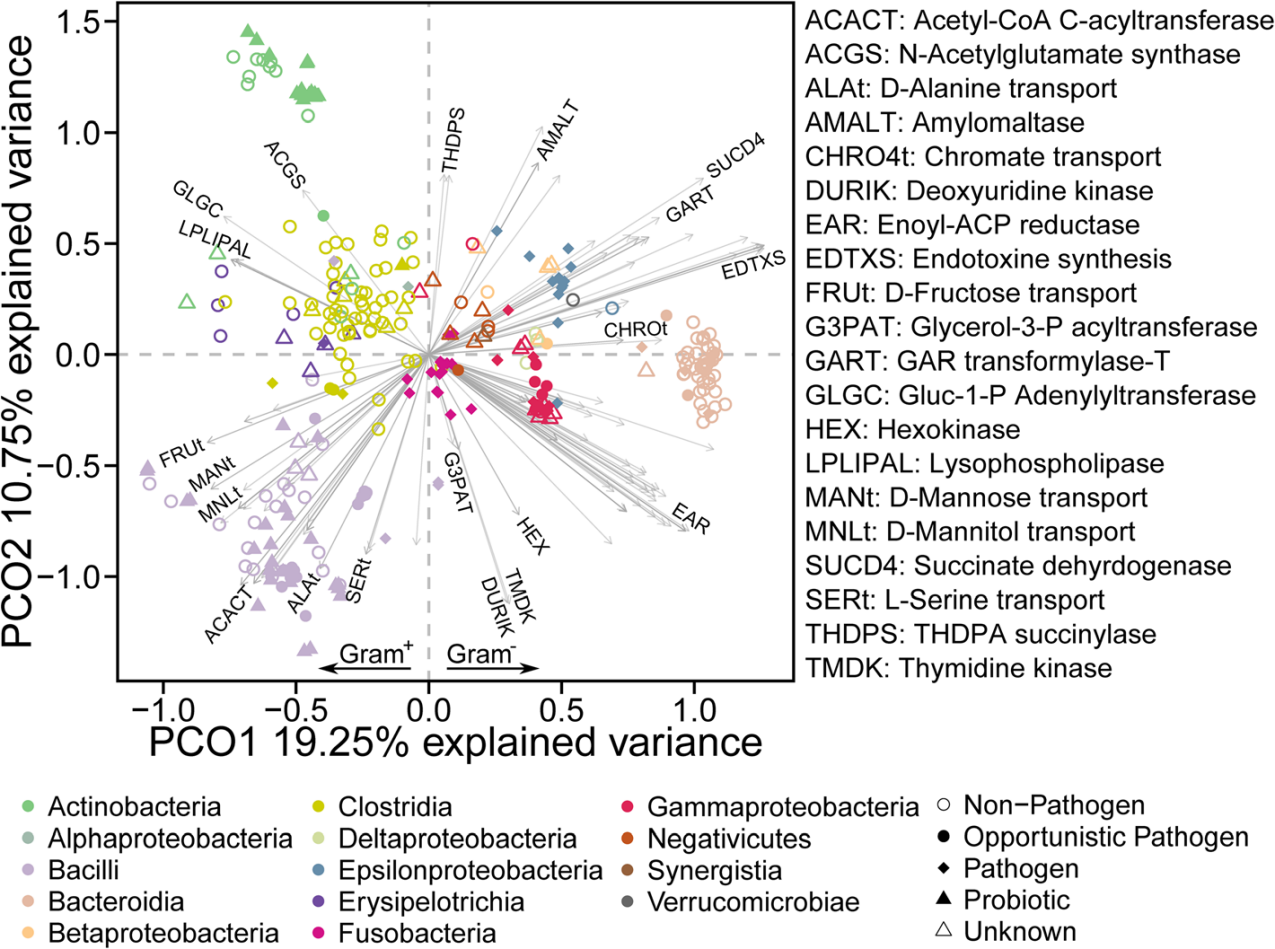
Global differences within metabolic models and their most divergent reactions. Biplot of the principle coordinate analysis based on the metabolic distance determined by the presence/absence of specific reactions in the metabolic models. Taxonomic groups are represented by different *colors*. The 200 reactions most associated with the point separation are indicated as *arrows* pointing from the coordinate origin to the contributing direction. The *arrow shading* represents reactions overlapping in their direction of contribution. The complete set of 2272 reactions sorted by their relevance can be found in Additional file 4: Table S3

To assess the differences within the metabolic reconstructions, we tested whether they could recapitulate the taxonomy of the studied microbes. We therefore computed a metabolic distance between the reconstructions based on the reaction presence [32] and subsequently used principle coordinate analysis (PCoA) [33]. This analysis revealed clusters, which correspond to known taxonomic groups (Fig. 2). More specifically, with more than 30 % of explained variance, the first principle coordinate (Fig. 2) was able to discriminate between Gram-negative and Gram-positive bacteria, which is in concordance to traditional measures of broad taxonomic groups, assigned based on the phylogeny of the 16S rRNA gene, the production of fatty acids, and corresponding membrane lipid composition [34]. In our PCoA (Fig. 2), members of the class Negativicutes were closely associated with Gram-negative bacteria rather than their phylogenetically close Gram-positive relatives, which is in accordance to their unusual membrane composition including two membrane layers [35].

The separation between Gammaproteobacteria and Actinobacteria highlights that our reconstructions captured taxa-specific metabolic features, despite the mentioned annotation bias. Furthermore, Clostridia species showed a high metabolic diversity and overlapped with clusters of other microbial taxa (Fig. 2), which is consistent with the reported metabolic variety of these bacteria and their corresponding beneficial traits in the human gut [36]. Erysipelotrichia representatives are closely but nonetheless distinctly placed relative to the Clostridia in the 2D principle coordinate plot (Fig. 2). Intriguingly, members of Erysipelotrichia were formerly considered as Clostridia based on the phylogeny of marker genes [37] but then re-assigned to a novel class based on their phylogeny and membrane composition [38]. Similar to the Clostridia, Bacilli species were also widely spread in the 2D principle coordinate plot (Fig. 2), reflecting their metabolic versatility [39]. In contrast, other taxa had more dense clusters, particularly Actinobacteria, reflecting more specialized roles of these bacteria, such as the conversion of polysaccharides [40].

Overall, we propose that metabolic reconstructions could be used, in addition to canonical approaches, to assist in the taxonomic definition of novel microbes and the re-assignment of already described microbes into better defined taxonomic groups. In particular, our approach has the advantage of considering functional characteristics, in contrast to methods solely relying on the presence and phylogeny of marker genes. As also pointed out by previous studies [41], functional repertoires can have a positive influence on the annotation quality of taxonomic groups. Ultimately, this could shed light onto the metabolic versatility of microbes in general or in specific habitats, such as the human gut.

### Energy and membrane metabolism as markers for metabolic divergence

Following the broader characterization, we aimed to obtain a better understanding of the reactions driving the observed separation in the first two coordinates. The separation of taxonomic groups is due to reactions involved in membrane synthesis and central metabolism (Fig. 2). In particular, different types of lysophosolipase reactions exhibit the highest explanatory power (Additional file 4: Table S3). These reactions convert various phospholipid precursors (differing in their number of C-atoms) and have the same direction in the first principle coordinate, because all reactions can be carried out by single enzymes and are thus linearly dependent. Similarly, the amylomaltases catalyze multiple reactions differing in their substrates (Additional file 4: Table S3). For the enoyl-ACP reductase, we found a variety of reactions with different directions toward the first principle coordinate (Fig. 2). This variation in angle represents a potential variation in distinct yet convergent fatty acid synthesis processes involved in energy metabolism and known to be present in the human gut microbiome [2], thus contributing to the discrimination of the different types of bacteria. This observation is consistent with the fact that fatty acid profiles have been used to characterize microbial communities before the advent of nucleic acid-based methods [42]. The synthesis of endotoxins was positively associated with the distribution of Gram-negative pathogenic species within the Proteobacteria and Fusobacteria, which is in accordance with previously reported correlations between various diseases and the abundance of Proteobacteria-producing endotoxins [43]. The transport and utilization of diverse carbohydrates involved in energy metabolism, such as mannitol, mannose, and fructose, were positively associated with the location of the Bacilli cluster in the 2D principle coordinate plot (Fig. 2). This association highlights the variety of substrate consumption as represented by these reconstructions of microbial metabolism. In accordance with the literature, Bacilli are known to utilize a broad range of carbohydrates [44].

The differentiation of taxonomic groups based on reactions involved in energy and membrane metabolism may have important implications in understanding the evolution and heterogeneity of intestinal microbes. For instance, Gram-negative bacteria have been reported to change their membrane composition [45] in order to cope with environmental influences, such as antibiotics and human immune agents, many of which target bacterial membrane compounds [46]. Additionally, ecological changes within the microbial community [47] can provoke a differentiation in metabolic capabilities involved in energy metabolism leading to altered interactions of the community with the human host, supporting the observed high explanatory power of metabolic reactions toward cluster separation.

### The relationship between genotype, phenotype, and metabolic repertoire is non-linear

**Fig. 3:**
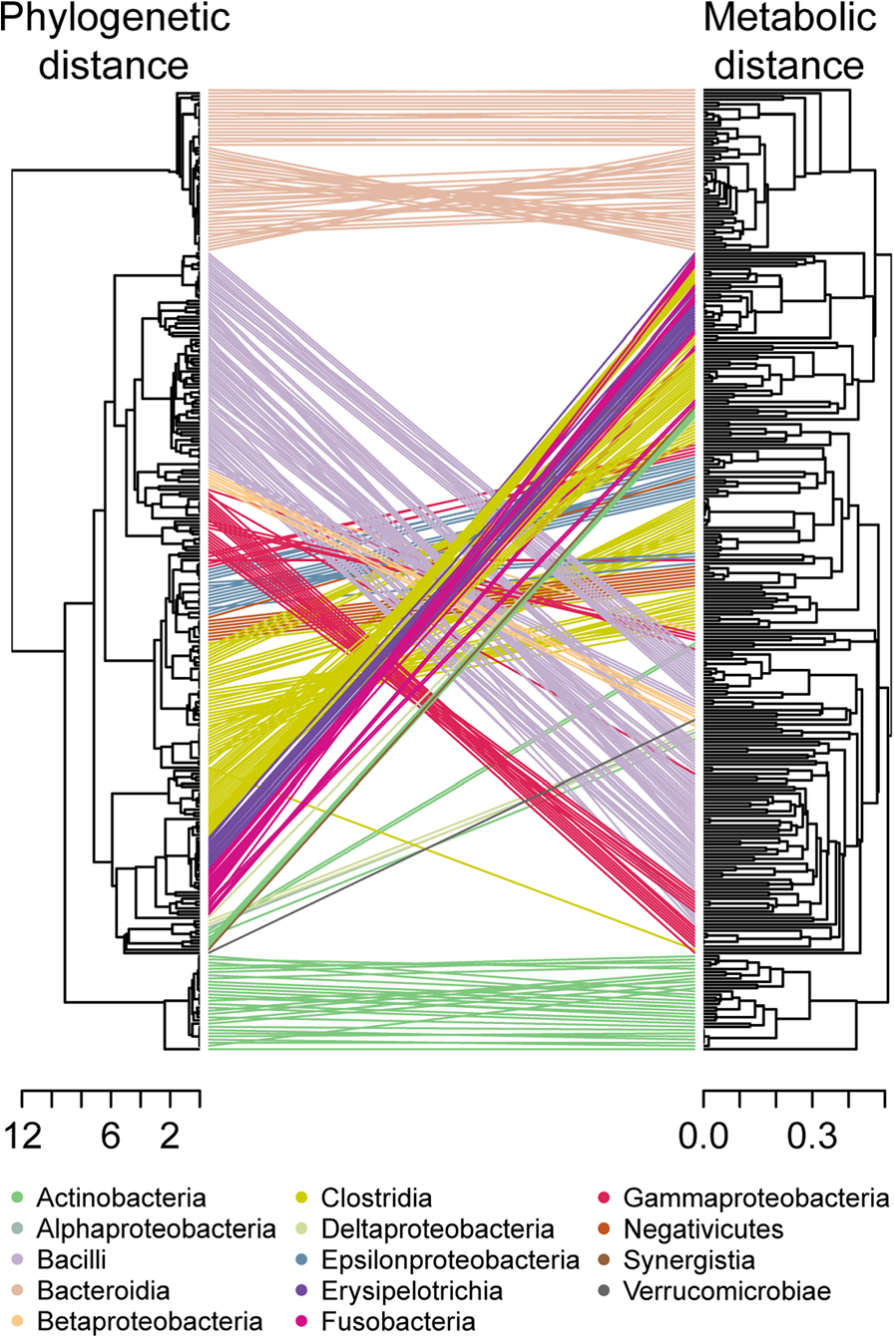
Tanglegram between the hierarchical clustering of the phylogenetic and metabolic distance. Tanglegram between the dendrograms of the reaction distance according to the presence of specific reactions and the phylogenetic distance according to the cophenetic distance of the maximum likelihood tree (rooted with two methanogenic archea) calculated from the sequence similarity of 400 selected essential genes. The dendrograms were calculated using hierarchical clustering with complete linkage. *Lines* connecting the same microbe are *colored* according to the taxonomic class

**Fig. 4:**
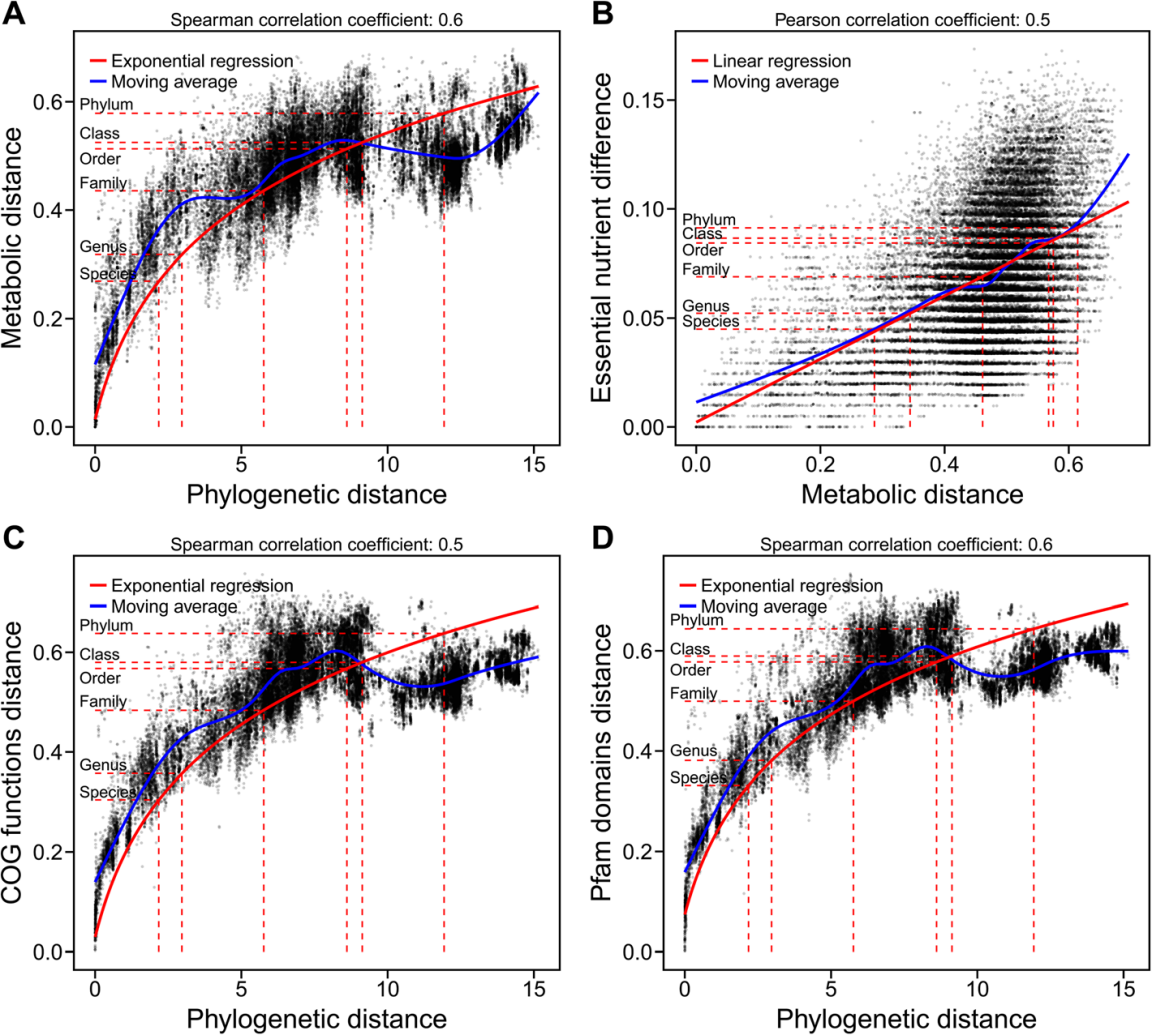
Relationship between reaction content, phylogeny, and phenotype. The metabolic distance was determined according to the presence of specific reactions in the model (A,B). COG (C) and Pfam (D) functional differences were assessed by comparing the presence/absence of COG functions and Pfam domains for all genomes, respectively. The phylogenetic distance is based on the cophenetic distance of the maximum likelihood tree (rooted with two methanogenic archea) calculated from the sequence similarity of 400 selected essential genes (**a**, **c**, **d**). The phenotype divergence was represented by the difference in essential nutrients, which were determined by removing the nutrient of interest from the in silico medium and subsequently checking for growth/no growth with flux balance analysis (**b**). The *shading* of the points (**a**–**d**) represents the density of all pairwise comparisons between the microbe models (*n* = 45.150). The *blue line* (**a**–**d**) represents the moving average over the data points. The goodness of fit for both regression models (**a**, **b**) can be found in Table 2. The means of the phylogenetic (**a**, **c**, **d**) and metabolic distances (**b**) for each taxonomic category are indicated by *dashed red lines*

To further investigate the observed metabolic diversity (Fig. 2) and its evolutionary basis, we computed the phylogenetic relationship between the 301 bacteria based on 400 protein-coding metabolic genes [48] using two methanogenic archaea as outgroups (Additional file 5: Figure S2). On the basis of this rooted phylogenetic tree, we computed pairwise phylogenetic distances from the heights within the tree using the cophenetic distance [49]. While the clustering of this phylogenetic distance (Fig. 3) recapitulated the original phylogeny (Additional file 5: Figure S2), we additionally computed a genetic distance based on the 16S rRNA gene similarity of the microbes (Additional file 6: Figure S3), to ensure that our observations were reproducible with other methods or markers. The pairwise distance based on the phylogenetic tree and the inferred presence of distinct reactions were overall congruent with each other (Fig. 3). Interestingly, we identified an exponential relationship between phylogeny and metabolic repertoire (Fig. 4), which is in accordance to a previous study based on genomic measures [50]. To exclude potential artifacts resulting from homology-based annotation methods (Model SEED) used for the generation of the metabolic reconstructions, we also determined the distance based on the presence of detected clusters of orthologous groups (COGs) [51] and Pfam protein domains [52]. These two measures also exhibited the same exponential relationship between metabolic repertoire and phylogeny (Fig. 4). Importantly, this relationship indicates that closely related species can have an extremely divergent set of metabolic reactions, while at taxonomic ranks above the family level, only limited amounts of additional emergent features were observed. Since COG annotations and Pfam domains are prone to misclassification, we also included annotation measures with a higher quality, such as MetaCyc functionalities [53] as well as EC numbers (Additional file 7: Figure S4) and observed a comparable exponential trend. Similar observations have been obtained in published experimental studies based on the phenotypic properties of different strains from the same genus or species [6, 8], underlining the biological relevance of our observations. In the context of a microbial community or biofilm, our observed relationship explains why closely related taxonomic groups (e.g., species of the same genus) are able to co-exist, while the overall consortium is limited in its metabolic potential [54]. In addition to this result, we identified a linear relationship between the metabolic repertoire and the similarity of essential nutrients, which we calculated using flux balance analysis as a proxy for the metabolic phenotype (Fig. 4b). These findings complement previous knowledge about the relationship between genotype and phenotype by Plata et al. [55]. Here, a similar exponential relation was observed between microbial phylogeny and varying growth conditions in selected genome-scale metabolic models, which were not directly associated with a specific habitat [55]. Additionally, this relationship has also been found with respect to the phenotypic similarity based on gene essentiality and synthetic lethal genes [55]. Taking into account that these latter measures have been based on flux balance analysis and are thus analogous to our results, we conclude that the observed patterns are generally applicable to bacteria. Furthermore, we argue that the metabolic network constituting of a set of reactions is appropriate to represent and explain a phenotype (Fig. 4b). Assuming the metabolic repertoire as one of the major factors for the evolution of intestinal microbes, transfer of metabolic traits within different taxa may account for fast metabolic diversification of species and strains leading to niche partitioning. In fact, horizontal gene transfer has been shown to be enriched within organisms inhabiting the same environment, particularly, the human gut [56]. In addition to the results of Plata et al. [55], we propose the metabolic repertoire as one of the major factors influencing the phenotypic differentiation of human gut microbial communities. Still, the clear separation of taxonomic groups noted above (Fig. 2) suggests that exchange of functionalities is limited to ensure a certain metabolic divergence within the whole microbiota to maintain functional diversity and limit competition between closely related organisms.

### The relationship between phylogeny, metabolic repertoire, and phenotype is taxon-dependent

**Fig. 5:**
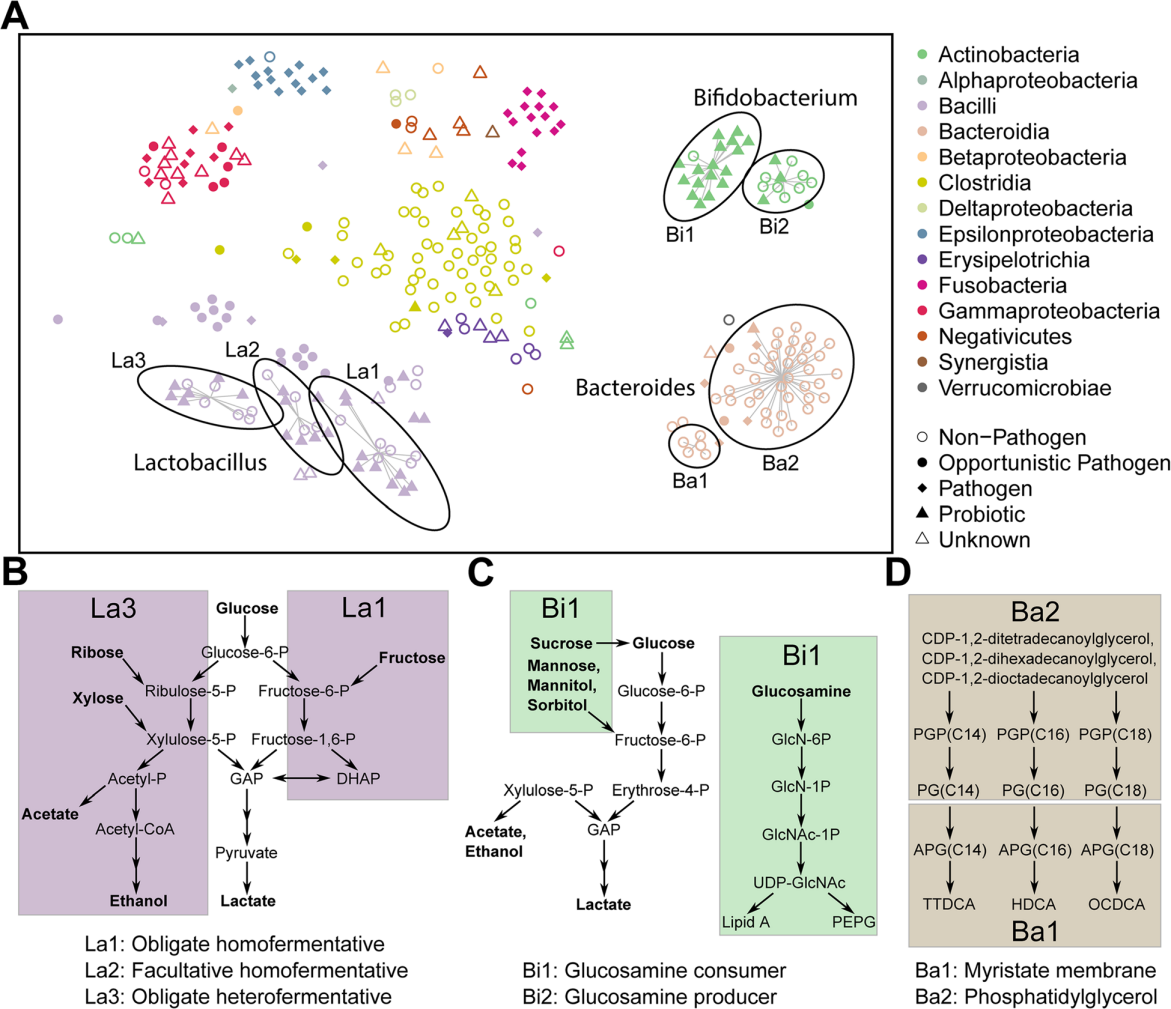
Local differences within metabolic models and their sub-type-specific pathways. The metabolic distance was determined according to the presence of specific reactions in the model (**a**). t-SNE was performed to obtain a low dimensional representation of the local differences within taxonomic groups which are represented by the different *colors*. Sub-types are defined based on hierarchical clustering of the reaction similarities. Members of one sub-type are connected with *lines* which originate from the cluster centroid. The *ellipses* represent confidence intervals of the clusters with a certainty of 95 %. Distinguished pathways within sub-types include the genera *Lactobacillus* (**b**), *Bifidobacterium* (**c**), and *Bacteroides* (**d**). The pathways occurring in only one of the sub-types are framed by *boxes* carrying the corresponding cluster name. Reactions within pathways are represented by *black arrows. GAP* glycerol-3-phosphate, *PEPG* peptidoglycan, *PGP* phosphatidylglycerophosphate, *PG* phosphatidylglycerol, *APG* 1-Acyl-sn-glycero-3-phosphoglycerol, *TTDCA* tetradecanoate, *HDCA* heptadecanoate, *OCDCA* octadecanoate

To account for taxon-dependent differences between microorganisms (Table 1), we focused our analysis on model subsets of the five classes and the three genera with the highest number of representatives (Table 2). Additionally, this focus allows us to elucidate whether our results were dependent on our selection of microbes or could be expanded to other microbes not considered in this study. We found that the exponential relationship between phylogeny and metabolic repertoire as well as the linear relationship between nutrient essentiality and metabolic repertoire was apparent for most taxonomic groups (Table 2). However, we noticed differences within the taxa. In particular, there was a considerable exponential fit for all five major bacterial classes except for Clostridia, which could be explained by Clostridia’s broad metabolic versatility and the corresponding difficulties in the taxonomic assignment within this class [57]. Our result is in accordance with the observed cluster variability of Clostridia when comparing the clustering of the metabolic and phylogenetic distance (principal coordinate analysis, Fig. 3). When investigating individual genera, we detected a high correlation between essential nutrients and the metabolic repertoire of *Bifidobacteria*, whereas the correlation between their phylogeny and metabolic repertoire was less pronounced (Table 2, Fig. 3). For members of the genus *Bacteroides*, the metabolic repertoire correlated strongly with their phylogeny (Fig. 3), but only weakly with the essential nutrients (Table 2). Based on these results, we propose that the divergence within this genus can be explained by divergence in metabolic pathways relating to membrane synthesis (Fig. 5) rather than energy metabolism and thus nutrient essentiality. For the *Lactobacillus* genus, we found a strong correlation between metabolic potential with both, phylogeny and essential nutrients. Within this genus, energy metabolism explained particular phenotypic divergences of species (Fig. 5), which is consistent with the observed high correlation between reactions involved in nutrient uptake and the clustering of representatives of the Bacilli in the principal coordinate plot (Fig. 2). Taken together, our results show a generality of the observed relationships between phylogeny, metabolic repertoire, and nutrient essentiality within and between taxonomic groups (Fig. 4).

**Table 2:** Summary statistics of the relationship between reaction content, phylogeny, and essential nutrients

	Reaction/phylogeny			Reaction/essential nutrients		
	(exponential model^a^)			(linear model)		
Taxon considered	Spearman correlation	*R* ^2^	RMSE	Pearson correlation	*R* ^2^	RMSE
All taxa	0.59^b^	0.62^b^	0.11	0.48	0.23	0.03
Class						
Bacilli	0.68^b^	0.69^b^	0.08	0.58^b^	0.34	0.02
Clostridia	0.61^b^	0.39	0.12	0.67^b^	0.45	0.02
Bacteroidia	0.90^b^	0.80^b^	0.06	0.62^b^	0.38	0.02
Actinobacteria	0.80^b^	0.76^b^	0.16	0.86^b^	0.74^b^	0.02
γ-Proteobacteria	0.78^b^	0.56^b^	0.11	0.72^b^	0.52^b^	0.01
Genus						
*Lactobacillus*	0.75^b^	0.70^b^	0.08	0.56^b^	0.31	0.03
*Bifidobacterium*	0.42	0.29	0.07	0.83^b^	0.69^b^	0.01
*Bacteroides*	0.81^b^	0.79^b^	0.05	0.33	0.11	0.02

^a^The exponential model was represented by a linear regression of semi-logarithmic transformed data

^b^Values above 0.5

### Reaction differences reflect metabolic versatility among closely related microbes

To further investigate the metabolic divergence within closely related microbes of the same taxonomic group, we used t-distributed stochastic neighbor embedding (t-SNE) [58] for the two-dimensional visualization of the reaction similarities (Fig. 5, see Additional file 8: Figure S5 for point labels). t-SNE is a non-linear, non-parametric dimensionality reduction and has been used previously to reveal data-inherent cluster structures [59–61]. This method enabled us to identify fine-scale reaction differences, in addition to the principal coordinate analysis (Fig. 2). Several distinct clusters were apparent and corresponded to the different bacterial classes (Fig. 5). We further focused our analysis on the three most abundant genera in our model selection (Table 1). For *Lactobacillus*, we noted a widespread metabolic repertoire and thus a relatively large variability of members within this group (Fig. 5). We identified three distinct subclusters (La1, La2, and La3) within this genus. While La2 showed major overlaps with the other two clusters, La1 and La3 were distinct from each other. We investigated the differences in the reaction sets between the representatives of the different subclusters (Additional file 9: Table S4). Based on the present reaction sets, La1 corresponds to obligate homofermentative La2 to facultative homofermentative and La3 to obligate heterofermentative pathways involved in the energy metabolism of lactic acid bacteria (Fig. 5b). The pathway presence in the genomes explains why La2 overlaps with the other clusters, since the facultative homofermentative group (La2) shares reactions with the obligate homofermentative (La1) and heterofermentative group (La3) [44]. In agreement with the literature, these subclusters correspond to known divergent pathways involved in energy metabolism in *Lactobacilli* [39]. This distinction of biologically relevant phenotypic groups using predicted difference in metabolic reactions encouraged us to propose novel bacterial sub-types. Therefore, we confirmed for our choice of the number of subclusters by performing hierarchical clustering (Fig. 3) to ensure that the subclusters were substantially different. For the *Bifidobacteria*, we propose two distinct subclusters (Bi1 and Bi2), which differed in the reactions involved in energy metabolism and membrane biosynthesis (Fig. 5c). For the energy metabolism, numerous reactions involved in the uptake and utilization of diverse carbohydrates were observed for members of the subcluster Bi1 (Additional file 11: Table S5), corresponding to known strain-specific differences within closely related *Bifidobacteria* [62]. Furthermore, we found reactions involved in the uptake and conversion of glucosamine to peptidoglycan, which could be associated with membrane composition in these two groups. To our knowledge, such pathway differentiation has not yet been proposed for *Bifidobacteria*. For the *Bacteroidia*, we could distinguish two subclusters (Ba1 and Ba2). The differences between these clusters can be attributed to the membrane biosynthesis (Fig. 5d; Additional file 12: Table S6). Members of Ba2 possess various pathway types leading to the production of varying phosphatidylglycerol compounds, whereas members of Ba1 can further process phosphatidylglycerol to myristic acid. This finding is of particular biological importance, when considering the virulence and signaling purposes of membrane lipids in *Bacteroides* species found in previous studies [63, 64], which links the phenotype to the synthesis of membrane compounds. Furthermore, since energy metabolism and substrate availability via the diet are major ecological driving forces within the human gut microbiota [2], the metabolic diversification of other closely related microbes, such as *Lactobacillus* spp. and *Bifidobacterium* spp., can be a necessary requirement to maintain a stable coexistence with each other and the host. Considering that optimal conditions for metabolic cooperation are dependent on the similarity between the metabolic repertoires of several species [32], this pathway analysis approach could be used to estimate cooperative as well as competitive strategies. In particular, microorganisms tend to have a higher cooperativity if they are not too similar nor too different [65], indicating that members of the same taxon, but different subclusters (Fig. 5b) might be able to co-exist, whereas functionally similar microbes may be more likely to compete with each other [54].

## Conclusions

The requirement for a certain functional diversity to ensure a well-functioning cooperative intestinal microbiota is crucial to break down various complex dietary compounds and divide metabolic tasks among different community members [66]. Our results complement these ideas by investigating the metabolic divergence within a model microbiota, which can be primarily distinguished by reactions involved in energy and membrane metabolism. These capabilities play important roles in shaping the interface between host and symbionts, and thereby may lead to a deeper understanding in addition to metagenomic analyses in which all microbial functions are assessed [1]. Furthermore, the metabolic repertoire of microbes is proportional to their phenotypic properties, highlighting the importance of diversity in explaining the metabolic processes taking place within the human gut. In contrast to these properties, the metabolic repertoire exhibited an exponential relationship with phylogeny, underlining the challenges in inferring metabolic functions from phylogeny alone, in particular when using single gene-centric approaches such as via 16S rRNA gene amplicon sequencing. Moreover, this circumstance can be regarded as an important evolutionary and ecological feature of the microbiome; functional components constituting whole pathways can be very different within closely related species, whereas the metabolism in the overall metabolic repertoire is limited. In other words, by dividing the metabolic tasks between certain taxonomic groups, the microbiota can make efficient use out of a small set of functions thereby facilitating niche partitioning. This result has important implications when considering the overall species richness of the human gut microbiome in the context of different patients and diseases [67]. Further analyses could prove these concepts by modeling interactions within bacteria and the use of the here reconstructed and refined genome-scale metabolic models.

## Methods

### Metabolic model selection, construction, and refinement

We selected a set of 301 microbes (Additional file 3: Table S1) representing species present in the normal gut microbiota of healthy individuals, according to previous studies [1, 3]. We retrieved the genome sequences as well as additional information about the sequencing status, oxygen requirement, taxonomic placement, and phenotype from the integrated microbial genome database [68]. The completeness and possible genomic contamination by other microorganisms of the individual 301 genomes was assessed using a collection of 107 universal, single-copy genes [28]. The genomic sequences were uploaded for gene annotation to the RAST server [69] using default parameters. Draft metabolic reconstructions were then built with these genome annotations using the Model SEED pipeline [19]. To ensure, that the metabolic models are able to grow under anaerobic conditions, which are prevalent in their natural ecosystem, we modified, if necessary, one to five reactions to enable anaerobic growth. The reactions modified for each model are listed in Additional file 1: Table S2. For descriptive purposes, reactions in the metabolic models were translated into our in-house metabolite and reaction database. The original SEED reaction nomenclature was maintained for the growth simulation. All refined draft metabolic models are publically available in their Matlab format at http://thielelab.uni.lu/in_silico_models.

### Growth simulation

To compute different growth conditions, the metabolic reconstructions were subjected to flux balance analyses [10] with the COBRA Matlab toolbox [9] using IBM ILOG cplex as the linear programming solver (IBM, Inc.). Briefly, genome-scale metabolic models were represented as a stoichiometric matrix ***S***, which encodes information about the mass balance of the complete set of enzymatic and transport reactions as well as a biomass reaction. The biomass reaction was retrieved from the metabolic reconstructions and represents the production of cellular building blocks (e.g., cofactors, amino acids, and lipids). Based on the stoichiometry, we could distinguish in our set of models 17 distinct biomass reactions, and based on the qualitative presence of compounds, we could distinguish 6 types of distinct biomass reactions (Additional file 3: Table S1). Hence, the automatically included biomass reactions from the Model SEED pipeline are different and therefore reflect different precursor needs of the considered microbes. Given this reaction as an objective for the biological system, the metabolic fluxes of all reactions in steady-state maximizing growth can be determined by defining an optimization problem as follows:

$$ \begin{array}{c}\hfill \mathrm{maximize}\kern0.24em {v}_b\hfill \\ {}\hfill \mathrm{subject}\kern0.24em \mathrm{t}\mathrm{o}\kern0.24em \boldsymbol{S}\cdot v=0\hfill \\ {}\hfill {v}_{i, \min}\le {v}_i\le {v_i}_{, \max },\forall i\in n\kern0.24em \mathrm{reactions}\hfill \end{array} $$

With *v*_*b*_ as the flux through the biomass objective function, *v* as the vector of all reaction fluxes, *v*_*i*,*min*_ as the minimal flux capacity of reaction *i*, and *v*_*i*,*max*_ as the maximal flux capacity of reaction *i*. The solution (metabolic fluxes of all reactions) of this optimization problem can be obtained using linear programming. The flux through the biomass reactions can be interpreted as the growth rate of the microbe model. By setting the constraints *v*_*i*,*min*_ and *v*_*i*,*max*_ of exchange (transport) reactions, varying growth conditions can be simulated. Throughout this study, the maximal uptake was constrained to 10 mmol/gDW/h to estimate natural occurring conditions. The maximal achievable growth rate was calculated under these conditions by assuming that all exchange reactions are potentially active (equivalent to rich medium condition). Additionally, the absence of a particular metabolite in the medium was simulated by setting its minimal and maximal exchange reaction constraints (*v*_*i*,*min*_ and *v*_*i*,*max*_) to 0 mmol/gDW/h. By the iterative removal of each metabolite individually from the rich medium for each microbe model, different growth conditions were simulated. Essential nutrients were defined by growth rates smaller than 0.05 h^−1^ after removal from the medium. This cutoff was based on the estimated growth rate of microbes within the mammalian gut [70]; however, all calculated smaller growth rates were below 0.0001 h^−1^ and thus negligible.

### Data mining of metabolic and genomic information

To assess the differences between the individual microbes, we used the reaction content and essential nutrients as well as COG functions and Pfam domains. The reaction content was based on the metabolic models obtained from Model SEED [71], whereas the COG functions and Pfam domains were obtained from the integrated microbial genomes database [68]. For each microbe, the presence and absence of reactions, essential nutrients, and functions were assessed in relation to the union of all metabolic reconstruction and genome annotations, respectively. The resulting binary vector *b* was then analyzed between species *i* and *j* with the Jaccard Index as:

$$ \frac{b_i\cap {b}_j}{b_i\cup {b}_j} $$

to calculate the metabolic proximity according to [32]. Based on the obtained distance matrix of the reaction content, we used principle coordinate analysis [33] and t-SNE [58] for reducing the dimensionality from 301 to 2. The two-dimensional embeddings were visualized by scatter plots. Using principle coordinate analysis, we analyzed reaction differences between the metabolic models on a global scale by correlating each reaction to the principle coordinates and subsequently selecting the 200 reactions with the highest correlation (Additional file 4: Table S3). The t-SNE-based visualization was used to identify local differences, with a detailed analysis of cluster structures within the genera *Lactobacillus*, *Bifidobacteria*, and *Bacteroides*. The reaction set differences between the determined sub-types of these genera were then used to identify type specific pathways.

### Phylogenetic analysis

In addition to the determined metabolic difference, we used the phylogenetic relationships between the microbes as a measure of divergence. The phylogeny was computed with PhyloPhlAn, which uses a set of around 400 protein-coding genes for the phylogenetic placement [48]. In addition to the 301 bacterial genomes, the genomes of the archaea *Methanobrevibacter smithii* ATCC35061 and *Methanosphaera stadtmanae* DSM 3091 were used as an out-group to root the phylogenetic tree (Additional file 5: Figure S2). The resulting phylogenetic tree was visualized using EvolView [72]. The phylogenetic difference between the different bacteria was computed using the cophenetic distance based on the rooted tree [49].

### Correlation between phylogeny, metabolic repertoire, and essential nutrients

We determined the relationship between the metabolic repertoire of the models and the phylogenetic distance as well as its relation to the predicted essential nutrients by representing the phylogenetic distance as a function of the metabolic distance. We fitted different regression functions and found an exponential model defined by:

$$ y={10}^{\left(\alpha +\beta x\right)} $$

to be the most suitable for explaining the relationship between metabolic distance *x* and phylogenetic distance *y*. For the relationship between essential nutrient difference *z* and metabolic difference *x*, we found a linear model defined by:

$$ z=\alpha +\beta x $$

to be the best fit. We complemented the exponential model with the Spearman correlation and the linear model with the Pearson correlation as a measure of association between the variables. The goodness of fit measures for the different models and subsets of the data can be found in Table 2. The fitted parameters *α* and *β* for all plots in Fig. 4 can be found in Additional file 10: Table S7.

## Additional files

Additional file 1: Table S2. Table of the gap-filled reactions used to ensure anaerobic growth. (XLSX 14 kb) Additional file 2: Figure S1. Comparison between a set of our draft reconstructions and a set of published manually curated reconstructions. (TIFF 13914 kb) Additional file 3: Table S1. List of genome and model statistics of the microbe selection. (XLSX 94 kb) Additional file 4: Table S3. List of all reactions sorted according to their contribution to the point separation in Fig. 2. (XLSX 126 kb) Additional file 5: Figure S2. Phylogenetic maximum likelihood tree (rooted with two methanogenic archaea) calculated from the sequence similarity of 400 selected essential genes. (TIFF 13054 kb) Additional file 6: Figure S3. The exponential relationship between the phylogeny and reaction content using the 16S rRNA sequence similarity as a measure for genetic distance. (TIFF 3150 kb) Additional file 7: Figure S4. The correlation between MetaCyc and EC functionalities with the phylogenetic distance. (TIFF 13708 kb) Additional file 8: Figure S5. The same t-SNE-based, two-dimensional coordinates as in Fig. 5 with additional point labels for the different organisms. (TIFF 1771 kb) Additional file 9: Table S4. List of genera members belonging to the different clusters presented in Fig. 5. (XLSX 10 kb) Additional file 10: Table S7 The fitted parameters of the exponential models in Fig. 4. (XLSX 9 kb) Additional file 11: Table S5. Table with reaction differences within the clusters found for *Bifidobacterium*. (XLSX 30 kb) Additional file 12: Table S6. Table with reaction differences within the clusters found for *Bacteroides*. (XLSX 34 kb)
