# Erratum to: Phenotypic differentiation of gastrointestinal microbes is reflected in their encoded metabolic repertoires

**DOI:** 10.1186/s40168-016-0180-3

**Published:** 2016-07-04

**Authors:** Eugen Bauer, Cedric Christian Laczny, Stefania Magnusdottir, Paul Wilmes, Ines Thiele

**Affiliations:** Luxembourg Centre for Systems Biomedicine, University of Luxembourg, Esch-sur-Alzette, Luxembourg

## Erratum

Upon publication, a mistake in Additional file 2: Figure S1 was found in the original version of this article [1]. This mistake has been corrected in the below figure and did not result in any major difference in the authors’ conclusions.

## Additional file

Additional file 2: Figure S1. Comparison between a set of our draft reconstructions and a set of published manually curated reconstructions. (TIF 13.1 mb)
