# Supplementary figures and images for: Phenotypic differentiation of gastrointestinal microbes is reflected in their encoded metabolic repertoires
Source: Microbiome. 2015 Nov 30;3:55. doi: 10.1186/s40168-015-0121-6 (PMC4663747; doi:10.1186/s40168-015-0121-6)

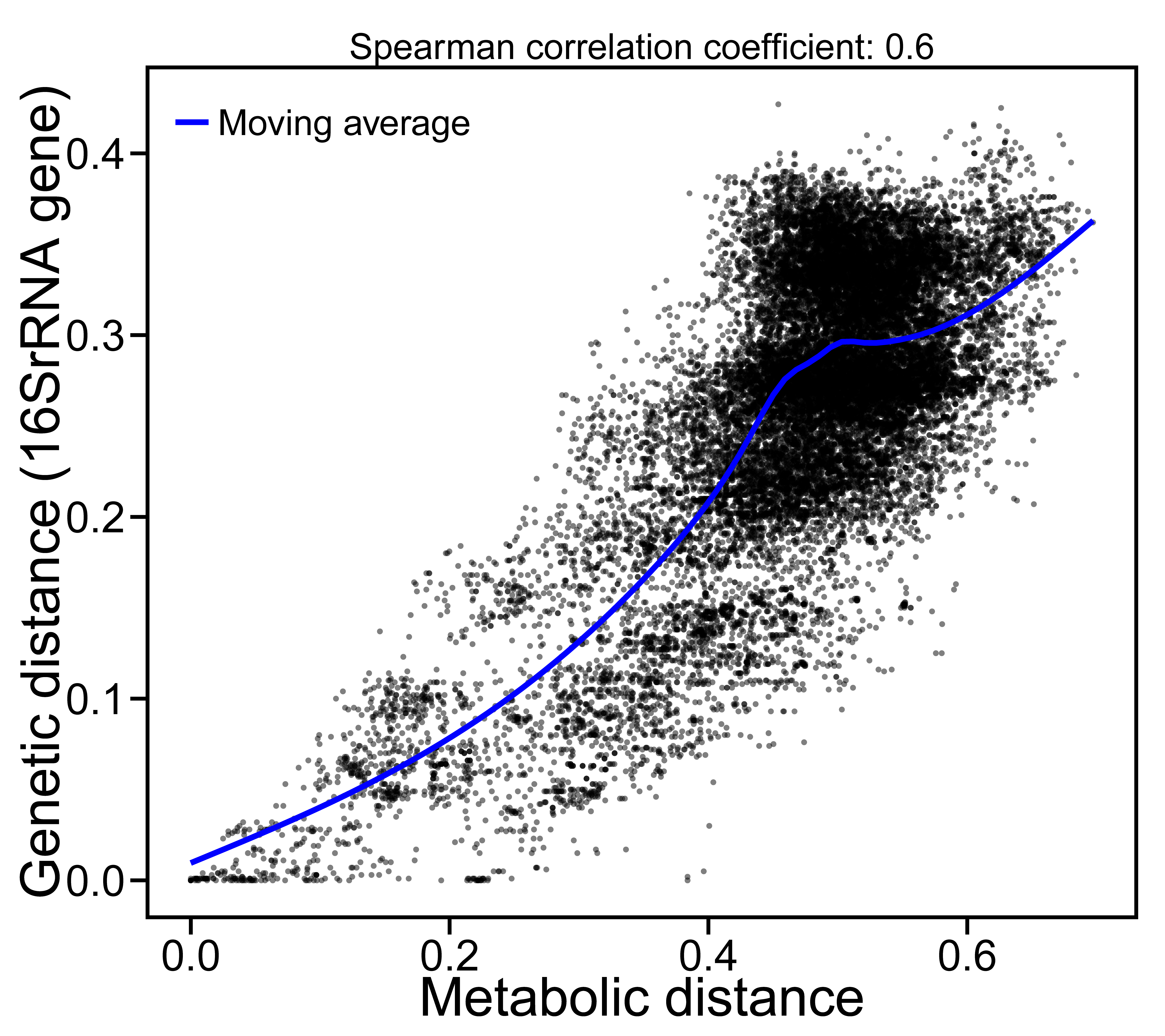

Supplement: Additional file 6: Figure S3. — The exponential relationship between the phylogeny and reaction content using the 16S rRNA sequence similarity as a measure for genetic distance. (TIFF 3150 kb) [file 40168_2015_121_MOESM6_ESM.tif]

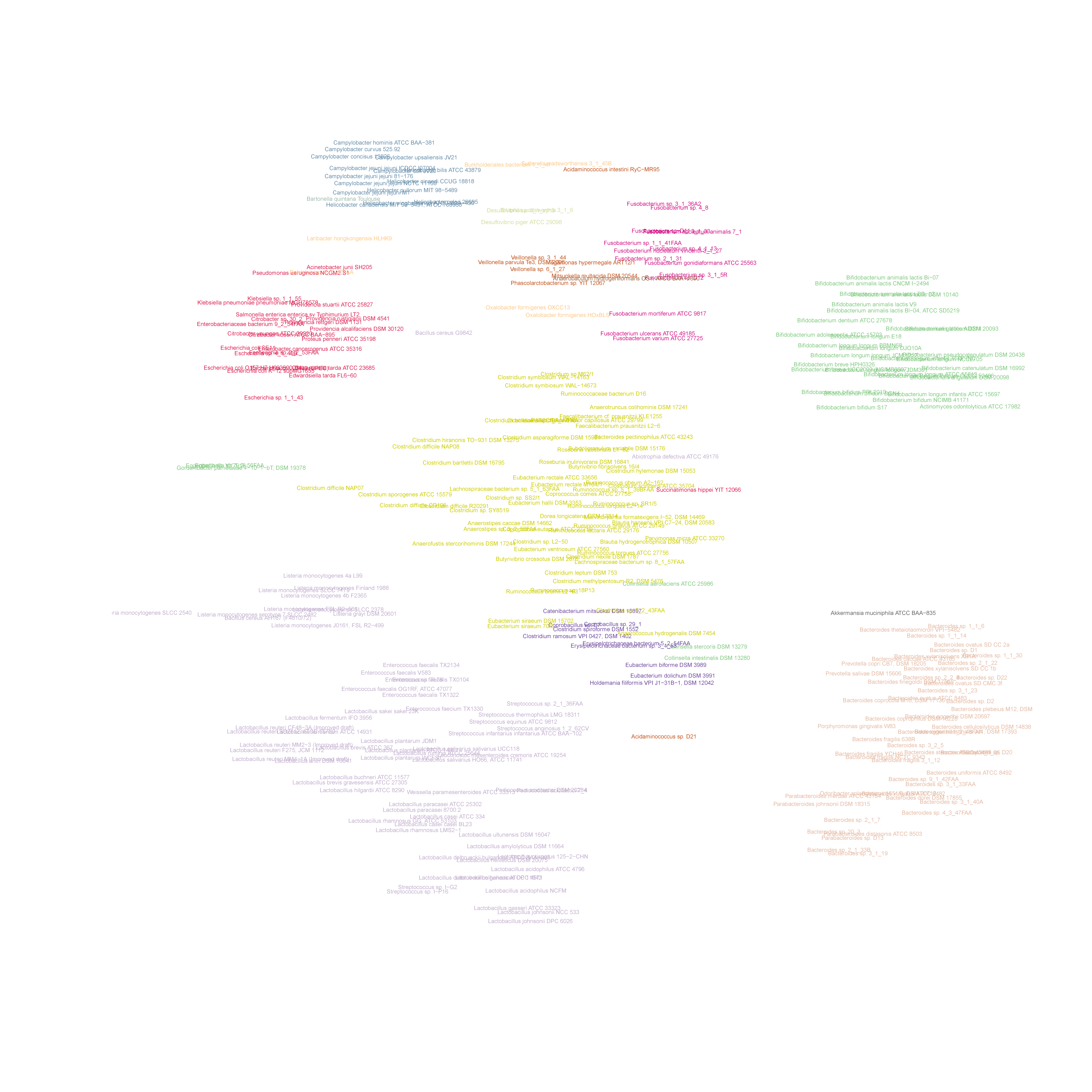

Supplement: Additional file 8: Figure S5. — The same t-SNE-based, two-dimensional coordinates as in Fig. 5 with additional point labels for the different organisms. (TIFF 1771 kb) [file 40168_2015_121_MOESM8_ESM.tif]
